# Vitamin D status and *Helicobacter pylori* infection: clinical associations and lipid pathway differences in an exploratory metabolomics sub-study

**DOI:** 10.3389/fnut.2026.1812874

**Published:** 2026-06-01

**Authors:** Said El Shamieh, Mai Abdel Jabbar, Rajaa Fakhoury, Ahmad Aljada, Najwa Anouti, Dima Kreidieh, Dalia Hassan, Mohamad Ali Hijazi, Hana M. A. Fakhoury

**Affiliations:** 1 Molecular Testing Laboratory, Department of Medical Laboratory Technology, Faculty of Health Sciences, Beirut Arab University, Beirut, Lebanon; 2 Department of Biochemistry and Molecular Medicine, College of Medicine, Alfaisal University, Riyadh, Saudi Arabia; 3 Department of Nutrition and Dietetics, Faculty of Health Sciences, Beirut Arab University, Beirut, Lebanon; 4 Pharmaceutical Sciences Department, Faculty of Pharmacy, Beirut Arab University, Beirut, Lebanon

**Keywords:** *Helicobacter pylori*, lipid metabolism, sphingolipid metabolism, untargeted metabolomics, vitamin D [25(OH)D]

## Abstract

**Background:**

Vitamin D has immunomodulatory functions, and lower serum levels have been associated with increased risk of chronic infections, including *Helicobacter pylori*. We evaluated the association between vitamin D status and *H. pylori* infection and explored vitamin D–associated metabolic differences in an exploratory untargeted metabolomics sub-study.

**Methods:**

This cross-sectional study enrolled 502 unrelated Lebanese adults (323 females, 179 males). Demographic, clinical, and biochemical variables were collected, including serum total 25-hydroxyvitamin D [25(OH)D] and serum iron levels. *H. pylori* infection was assessed by urea breath test or histology. Multivariable logistic regression was used to examine factors associated with *H. pylori* infection. Untargeted metabolomics was performed in an exploratory sub-study (*n* = 50) that included both *H. pylori*–positive and –negative participants, comparing vitamin D–deficient versus non-deficient individuals, followed by pathway enrichment analysis.

**Results:**

Vitamin D deficiency was more frequent among *H. pylori*–positive participants (*p* < 10^−23^), and mean serum iron level was lower among *H. pylori*–positive participants (78.39 μg/dL; *p* = 0.039). In multivariable analysis, compared with vitamin D deficiency, vitamin D insufficiency [OR = 0.11 (0.06–0.18), *p* = 2.58 × 10^−14^] and normal vitamin D status [OR = 0.02 (0.01–0.04), *p* = 4.35 × 10^−23^] were associated with lower odds of *H. pylori* infection; female sex was also associated with lower odds (OR = 0.44, 95% CI: 0.24–0.81; *p* = 0.01). In the metabolomics sub-study (*n* = 50), vitamin D deficiency was associated with differential metabolic features indicating altered lipid-related pathways, including sphingolipid (impact = 0.735; *p*_adj_ = 2.07 × 10^−6^), glycerophospholipid (impact = 0.468; *p*_adj_ = 0.005), and ether lipid metabolism (impact = 0.126; **p*_adj_* = 0.010).

**Conclusion:**

Vitamin D deficiency was strongly and dose-dependently associated with *H. pylori* infection in this cohort. In the metabolomics sub-study, vitamin D deficiency was associated with lipid pathway differences, most notably in sphingolipid metabolism. Longitudinal and mechanistic studies are warranted to clarify the temporal dynamics and biological significance of these lipid-metabolic signatures.

## Introduction

*Helicobacter pylori* is a major cause of chronic gastritis and peptic ulcer disease and is a recognized risk factor for gastric malignancy. Infection prevalence varies substantially across regions and socioeconomic settings and, although declining in many high-income countries, remains common in low- and middle-income settings (1). In the Middle East, infection rates remain high and have been reported to range between approximately 40.9 and 77.2% across studies, reflecting heterogeneity in populations and diagnostic methods (2).

In Lebanon, available evidence similarly indicates a persistent public health burden, with reported prevalence estimates differing by setting and diagnostic approach. A community-based cross-sectional study in Tripoli (2019–2020) reported 31% positivity by stool antigen testing among asymptomatic residents (3). In clinical settings, histology-based studies among symptomatic adults undergoing endoscopy in Beirut reported lower prevalence (e.g., 14.16%), whereas large hospital-based series have documented substantial seropositivity among dyspeptic patients in northern Lebanon (2). Beyond bacterial virulence factors, host susceptibility is shaped by demographic and environmental determinants including household crowding, sanitation, and socioeconomic conditions.

Vitamin D is increasingly recognized for its immunomodulatory roles, and hypovitaminosis D is common in Lebanon. In a large retrospective analysis of 19,452 Lebanese adults from Greater Beirut and Mount Lebanon conducted between 2013 and 2022, 12,604 (65%) were female and 6,848 (35%) were male; 31% were vitamin D deficient, 28% were insufficient, and 41% were sufficient (4). In parallel, iron deficiency and anemia have been associated with *H. pylori* infection in a systematic review and meta-analysis of 50 observational studies conducted across 19 countries, including populations from the Americas, Europe, Asia, Africa, and Oceania (pooled OR for iron deficiency anemia: 1.72, 95% CI 1.23–2.42; 14 studies), potentially through chronic gastritis and reduced gastric acidity that impair dietary iron absorption (5).

Mechanistically, vitamin D may influence host–pathogen interactions through lipid biology. Vitamin D signaling can interact with sphingolipid pathways, and alterations in ceramide and sphingolipid homeostasis have been linked to immune and inflammatory regulation (6). In *H. pylori* infection, host membrane cholesterol and cholesterol-dependent lipid microdomains have been implicated in bacterial persistence and immune modulation (7, 8), supporting the concept that host lipid remodeling may shape infection-related phenotypes. However, lipid-metabolic signatures associated with vitamin D status, and how these may intersect with *H. pylori* infection status, remain insufficiently characterized in human cohorts. Therefore, we aimed (i) to examine the association between vitamin D status and *H. pylori* infection in a Lebanese adult cohort and (ii) to explore, in an untargeted metabolomics sub-study, whether vitamin D deficiency is associated with lipid pathway differences in a subset that included both *H. pylori–* positive and negative participants.

## Methods

### Ethics approval and consent

The study protocol was approved by the Institutional Review Board of Beirut Arab University (BAU), Beirut, Lebanon (IRB no. 2019-H-0091-HS-R-0360). All participants provided written informed consent prior to participation.

### Study design, setting, and participants

This cross-sectional study was conducted in Lebanon and included 502 unrelated adults from the general population. Eligible participants were aged ≥18 years; no upper age limit was applied (oldest participant: 81 years). Individuals with a history of cancer or cardiovascular disease were excluded. Demographic, anthropometric, clinical, and lifestyle information was collected, including age, sex, marital status, smoking, physical activity, coffee consumption, and season of sampling (summer, autumn, winter, and spring). Although marital status was collected in the questionnaire, partners were not enrolled. Body mass index (BMI) was calculated as weight (kg)/height (m^2^) and categorized as normal (<25 kg/m^2^) or overweight/obese (≥25 kg/m^2^).

### Assessment of *Helicobacter pylori* infection

*Helicobacter pylori* infection status was determined using either the urea breath test (UBT) or histology. For UBT, testing was performed with a Heliprobe® BreathCard™ (Kibion GmbH, Germany), whereas histological assessment was performed on gastric biopsy specimens stained with hematoxylin and eosin (H&E), which were examined for the presence of *H. pylori* organisms using a brightfield light microscope at ×400 magnification.

### Biochemical measurements

#### Measurement of serum 25-hydroxyvitamin D [25(OH)D] levels

Serum total 25-hydroxyvitamin D [25(OH)D] levels were measured using the Elecsys™ Vitamin D total assay on the Roche platform (Roche Diagnostics, Basel, Switzerland), according to the manufacturer’s instructions. Vitamin D status was categorized according to the Endocrine Society Clinical Practice Guideline as deficiency (<20 ng/mL), insufficiency (≥20 to <30 ng/mL), and sufficiency/normal (≥30 ng/mL) (9). For the metabolomics sub-study, vitamin D status was dichotomized into deficient versus non-deficient (non-deficient = insufficiency + normal).

#### Measurement of serum iron and iron status

Serum iron level was quantified using the Roche IRON2 (Iron Gen. 2) kit on the Cobas c 111 chemistry analyzer (Roche Diagnostics, Basel, Switzerland), following the manufacturer’s instructions. Iron status was defined as deficient when serum iron level was <60 μg/dL; values ≥60 μg/dL were classified as normal, consistent with the dichotomous “iron status” variable used in regression analyses.

#### Exploratory metabolomics sub-study: design and grouping

An exploratory untargeted metabolomics analysis was conducted in a subset of 50 participants selected from the parent cohort, including 16 males and 34 females. Participants were selected based on sample availability and to ensure representation of both vitamin D-deficient and non-deficient groups, as well as both *H. pylori* infection statuses. The subset was not formally matched, and metabolomics comparisons were not adjusted for *H. pylori* infection status. For metabolomics analyses, participants were grouped by vitamin D status as deficient versus non-deficient, with the non-deficient group including participants with insufficient or normal vitamin D levels.

#### Metabolite extraction and sample preparation

Serum metabolites were extracted using an acetonitrile: methanol solvent mixture (1:1, v/v) added to serum at a 1:19 (serum: solvent) ratio. Quality control (QC) samples were prepared alongside study samples. Samples were incubated for 1 h at 25 °C on a thermomixer (600 rpm) and centrifuged at 16,000 rpm for 10 min at 4 °C. Supernatants were collected and dried using a SpeedVac concentrator. Dried extracts were reconstituted in 90 μL of a 1:1 (v/v) mixture of mobile phases A and B plus 10 μL internal standard solution, vortexed for 2 min, and centrifuged at 16,000 rpm for 10 min at 4 °C prior to LC–MS analysis.

Mobile phase A consisted of 0.1% formic acid in water. Mobile phase B consisted of 0.1% formic acid in methanol: acetonitrile (50:50, v/v).

#### Chromatographic separation

Reverse-phase separation was performed on an ACQUITY UPLC HSS T3 column (2.1 × 100 mm, 1.8 μm; 100 Å) maintained at 50 °C with a flow rate of 0.3 mL/min. The gradient started at 95% A/5% B and increased to a high organic content over the analytical run (as per the established laboratory LC method).

#### Mass spectrometry acquisition

Data were acquired in both positive and negative electrospray ionization modes using a Shimadzu LCMS-9030 quadrupole time-of-flight liquid chromatograph mass spectrometer (Q-TOF LC–MS/MS; Shimadzu Corporation, Kyoto, Japan) with external calibration. Acquisition consisted of a TOF-MS full scan (m/z 60–1,000; 100 ms) followed by data-independent MS/MS scans (m/z 40–1,000; 33 ms each) using 35 Da isolation windows and collision energy ramping from 5 to 55 V. A full cycle comprised 28 scans (~0.99 s). Full-scan TOF-MS data and MS/MS spectra were acquired for metabolite detection and annotation.

#### Data processing, annotation, and statistical analysis

Analyses were performed in R (version 4.4.2). Baseline characteristics were compared by *H. pylori* infection status using *χ*^2^ tests for categorical variables and independent-samples t tests for continuous variables. Multivariable logistic regression was used to evaluate factors associated with *H. pylori* infection, and results are reported as odds ratios (ORs) with 95% confidence intervals (CIs). Baseline characteristics are presented in Table 1. The multivariable model included the covariates presented in Table 2 (age category, sex, BMI category, marital status, smoking frequency categories, smoking duration, physical activity, coffee consumption, season of sampling, iron status, and vitamin D status). Statistical significance was set at *p* < 0.05.

**Table 1:** Baseline demographic, clinical, lifestyle, and biochemical characteristics of the study participants stratified by *Helicobacter pylori* infection status.

Characteristics	*Helicobacter pylori*			
	All patients	Positive	Negative	*p*
	(*n* = 502)	(*n* = 242)	(*n* = 260)	
	Mean ± SD	Mean ± SD	Mean ± SD	
Age (years)	39.93 ± 14.27	38.95 ± 13.94	40.85 ± 14.54	0.14
Sex *n* (%)				
Males	179 (36%)	95 (39%)	84 (32%)	0.13
Females	323 (64%)	147 (61%)	176 (68%)	
BMI categories *n* (%)				
Normal	257 (51%)	117 (48%)	140 (54%)	0.25
Overweight and obese	245 (49%)	125 (52%)	120 (46%)	
Marital status *n* (%)				
Not married	146 (29%)	74 (30%)	72 (28%)	0.022^*^
Married	335 (67%)	164 (68%)	171 (66%)	
Divorced	21 (4%)	4 (2%)	17 (6%)	
Cigarette consumption *n* (%)				
Non-smoker	368 (73.3%)	167 (69%)	201 (77%)	0.024^*^
1–10/day	39 (7.8%)	16 (7%)	23 (9%)	
11–20/day	65 (12.9%)	41 (17%)	24 (9%)	
More than 20/day	30 (6%)	18 (7%)	12 (5%)	
Intensity of physical activity *n* (%)				
None	64 (13%)	21 (9%)	43 (17%)	3.49 × 10^−5*^
Light	238 (47%)	141 (58%)	97 (37%)	
Moderate	154 (31%)	63 (26%)	91 (35%)	
Vigorous	46 (9%)	17 (7%)	29 (11%)	
Coffee consumption *n* (%)				
No	147 (29%)	77 (32%)	70 (27%)	0.27
Yes	355 (71%)	165 (68%)	190 (73%)	
Coffee frequency consumption (cups/day)	3.79 ± 4.12	3.42 ± 3.87	4.14 ± 4.32	0.05
Season of sampling *n* (%)				
Summer	128 (25%)	57 (24%)	71 (27%)	
Autumn	134 (27%)	66 (27%)	68 (26%)	
Winter	109 (22%)	53 (22%)	56 (22%)	0.80
Spring	131 (26%)	66 (27%)	65 (25%)	
Iron level (μg/dL)	81.81 ± 35.92	78.39 ± 33.94	84.99 ± 37.44	0.039^*^
Serum 25(OH)D (ng/mL)	24.06 ± 13.65	17.95 ± 7.46	29.71 ± 15.55	2.74 × 10^−24*^

Values are presented as *n* (%), or mean ± standard deviation (SD), as appropriate. *p* values were calculated using the *χ*^2^ test for categorical variables and the independent-samples *t* test for continuous variables. ^*^p < 0.05.

**Table 2:** Multivariable logistic regression analysis of demographic, lifestyle, and biochemical factors associated with *Helicobacter pylori* infection.

Characteristics	*Helicobacter pylori*		
	OR	95% CI	*p*
Age			
<40 years	1		
≥40 years	0.90	(0.48–1.70)	0.75
Sex			
Males	1		
Females	0.44	(0.24–0.81)	0.01*
BMI categories			
Normal	1		
Overweight and obese	1.25	(0.69–2.24)	0.46
Marital status			
Not married	1		
Married	1.16	(0.64–2.11)	0.62
Divorced	0.39	(0.09–1.53)	0.20
Cigarette frequency consumption			
Non-smoker	1		
1–10/day	0.65	(0.16–2.46)	0.53
11–20/day	2.12	(0.68–6.85)	0.20
More than 20/day	5.69	(1.07–35.53)	0.05
Period of smoking (years)	0.98	(0.93–1.04)	0.50
Physical activity			
No	1		
Yes	2.97	(0.99–9.07)	0.053
Coffee consumption			
No	1		
Yes	2.87	(1.40–6.02)	0.0045^*^
Season			
Summer	1		
Autumn	0.85	(0.47–1.56)	0.60
Winter	1.12	(0.63–2.01)	0.70
Spring	0.91	(0.50–1.65)	0.76
Iron status			
Deficiency	1		
Normal	0.95	(0.54–1.65)	0.85
Vitamin D status			
Deficiency	1		
Insufficiency	0.11	(0.06–0.18)	2.58 × 10^−14^*
Normal	0.02	(0.01–0.04)	4.35 × 10^−23^*

Odds ratios (ORs) and 95% confidence intervals (CIs) are shown. Reference categories were: age <40 years, male sex, normal BMI, not married, non-smoker, no physical activity, no coffee consumption, iron deficiency, and vitamin D deficiency. ^*^p < 0.05.

#### Metabolomics processing and analyses

Raw LC–MS data were processed using MZmine 2.53 for chromatogram deconvolution, peak detection, alignment, and feature extraction. Putative compound annotation was performed using MassBank, ChemSpider, HMDB, KEGG, and LipidMaps. Annotation confidence was assigned using the Metabolomics Standards Initiative (MSI) framework.

Downstream metabolomics analyses were performed using MetaboAnalyst 6.0. Prior to analysis, data were normalized, log-transformed, and Pareto-scaled. Differential abundance between vitamin D–deficient and non-deficient groups was assessed using moderated *t*-tests with Benjamini–Hochberg false discovery rate (FDR) correction (FDR < 0.05). Features were considered differentially abundant if they met both FDR significance and a fold-change threshold (≥1.5). Unsupervised and supervised multivariate analyses included Principal Component Analysis (PCA), Partial Least Squares Discriminant Analysis (PLS-DA), and Orthogonal Partial Least Squares Discriminant Analysis (OPLS-DA). These analyses were shown in Supplementary Figures 1 and 2. Pathway enrichment and topology analyses were conducted in MetaboAnalyst using the KEGG human pathway library, with adjusted *p*-values and pathway impact scores to prioritize pathways. ROC analysis was performed to evaluate the classification of vitamin D status using panels of increasing size (5, 10, 15, 25, 50, and 100 selected features); model performance was summarized using AUC with 95% CIs estimated via internal resampling.

## Results

### Participant characteristics by *Helicobacter pylori* infection status

Table 1 summarizes baseline characteristics stratified by *H. pylori* infection status. Mean age was comparable between *H. pylori*–positive and –negative groups (38.95 ± 13.94 vs. 40.85 ± 14.54 years; *p* = 0.14), and sex distribution did not differ significantly (*p* = 0.13). BMI categories were also similar between groups (*p* = 0.25). In contrast, marital status differed significantly by infection status (*p* = 0.022), with a higher proportion of divorced participants in the *H. pylori*–negative group. Smoking categories differed between groups (*p* = 0.024), driven by a higher proportion of *H. pylori*–positive participants in the 11–20 cigarettes/day category (17% vs. 9%); however, smoking duration in years did not differ (*p* = 0.60). Physical activity intensity differed significantly (*p* = 3.49 × 10^−5^), with light activity more prevalent among *H. pylori*–positive participants (58% vs. 37%), whereas the no-activity category was more common among *H. pylori*–negative participants (17% vs. 9%). This cross-sectional distribution does not indicate that sedentary behavior is protective against *H. pylori* infection, as the binary physical activity variable showed only a borderline association with infection in multivariable analysis (OR = 2.97, 95% CI: 0.99–9.07; *p* = 0.053; Table 2). The proportion of participants reporting coffee consumption did not differ significantly between groups (*p* = 0.27). However, coffee frequency tended to be higher among *H. pylori*–negative participants (4.14 ± 4.32 vs. 3.42 ± 3.87), although this difference was only borderline (p = 0.05). Mean serum iron level was lower in *H. pylori*–positive participants (78.39 ± 33.94 vs. 84.99 ± 37.44; *p* = 0.039), and serum 25(OH)D levels were substantially lower in *H. pylori*–positive compared with *H. pylori*–negative participants (17.95 ± 7.46 vs. 29.71 ± 15.55 ng/mL; *p* = 2.74 × 10^−24^).

### Factors associated with *Helicobacter pylori* infection in multivariable logistic regression

Table 2 presents the multivariable logistic regression analysis of factors associated with *H. pylori* infection. Age (≥40 vs. < 40 years; OR = 0.90, 95% CI: 0.48–1.70; *p* = 0.75), BMI category (overweight/obese vs. normal; OR = 1.25, 95% CI: 0.69–2.24; *p* = 0.46), marital status (married vs. not married; OR = 1.16, 95% CI: 0.64–2.11; *p* = 0.62; divorced vs. not married; OR = 0.39, 95% CI: 0.09–1.53; *p* = 0.20), smoking duration (years; OR = 0.98, 95% CI: 0.93–1.04; *p* = 0.50), and iron status (normal vs. deficiency; OR = 0.95, 95% CI: 0.54–1.65; *p* = 0.85) were not significantly associated with infection. Female sex was associated with lower odds of *H. pylori* infection than male sex (OR = 0.44, 95% CI: 0.24–0.81; *p* = 0.01). Among smoking categories, consumption of >20 cigarettes/day was associated with higher odds of infection compared with non-smokers (OR = 5.69, 95% CI: 1.07–35.53; *p* = 0.05), whereas other smoking categories were not significant. Coffee consumption (yes vs. no) was associated with higher odds of infection (OR = 2.87, 95% CI: 1.40–6.02; *p* = 0.0045). Physical activity (yes vs. no) was associated with a borderline increase in the odds of infection (OR = 2.97, 95% CI: 0.99–9.07; *p* = 0.053). Finally, compared with vitamin D deficiency (reference), vitamin D insufficiency (OR = 0.11, 95% CI: 0.06–0.18; *p* = 2.58 × 10^−14^) and normal vitamin D status (OR = 0.02, 95% CI: 0.01–0.04; *p* = 4.35 × 10^−23^) were associated with substantially lower odds of *H. pylori* infection.

### Exploratory untargeted metabolomics sub-study stratified by vitamin D status

**Figure 1:**
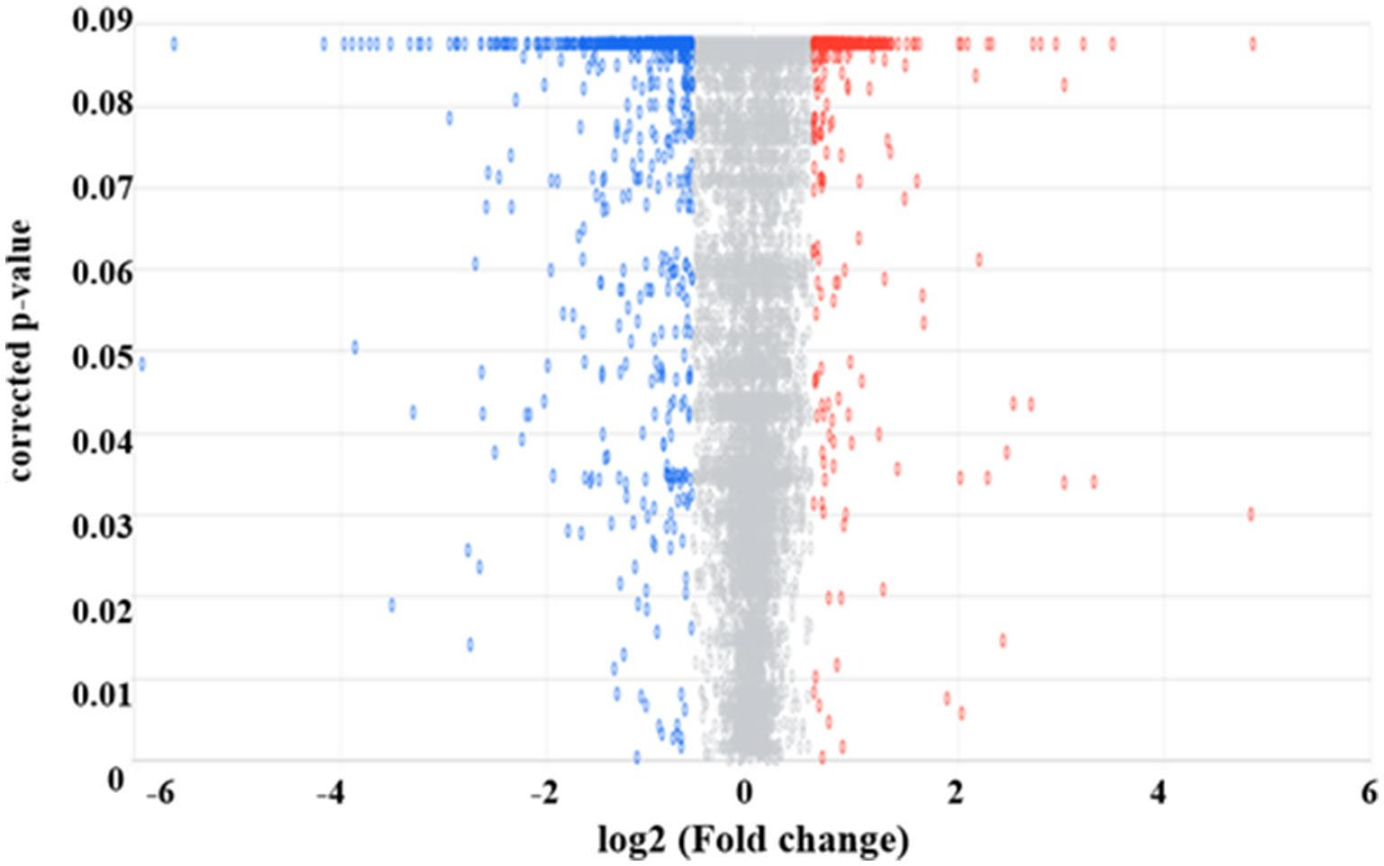
Volcano plot of untargeted LC–MS metabolic features associated with vitamin D status in the metabolomics sub-study (*n* = 50). The volcano plot shows differential abundance analysis of LC–MS molecular features comparing vitamin D–deficient versus non-deficient participants in the metabolomics sub-study (*n* = 50). The *x*-axis represents log_2_ fold change (vitamin D–deficient vs. non-deficient), and the *y*-axis represents the −log_10_ of the multiple-testing–corrected *p* value. Features meeting the predefined significance criteria (corrected *p* value threshold and fold-change cutoff) are highlighted as differentially abundant. Among 8,779 high-quality features, 799 were decreased (blue) and 422 were increased (red) in vitamin D–deficient sera, while the remaining 7,558 features were not significantly different between groups (gray).

We next performed an exploratory untargeted metabolomics analysis in the metabolomics sub-study (*n* = 50), which included both *H. pylori*–positive and –negative participants, comparing vitamin D–deficient versus non-deficient individuals (Supplementary Table 1). Because the metabolomics comparison was not adjusted for *H. pylori* status, the observed metabolic differences primarily reflect associations with vitamin D status in this mixed-infection subset. LC–MS data processing yielded 8,779 high-quality molecular features from 123,926 detected signals. Stratification by vitamin D status using volcano-plot analysis identified 1,221 differentially abundant features, with 422 increased and 799 decreased in vitamin D-deficient sera (Figure 1). Overall, a larger proportion of features were lower in the vitamin D-deficient group, suggesting broad metabolic differences associated with vitamin D status, particularly in lipid-related pathways such as sphingolipid and glycerophospholipid metabolism, as detailed in the pathway enrichment analysis below.

### Lipid pathway enrichment associated with vitamin D status in the metabolomics sub-study

**Figure 2:**
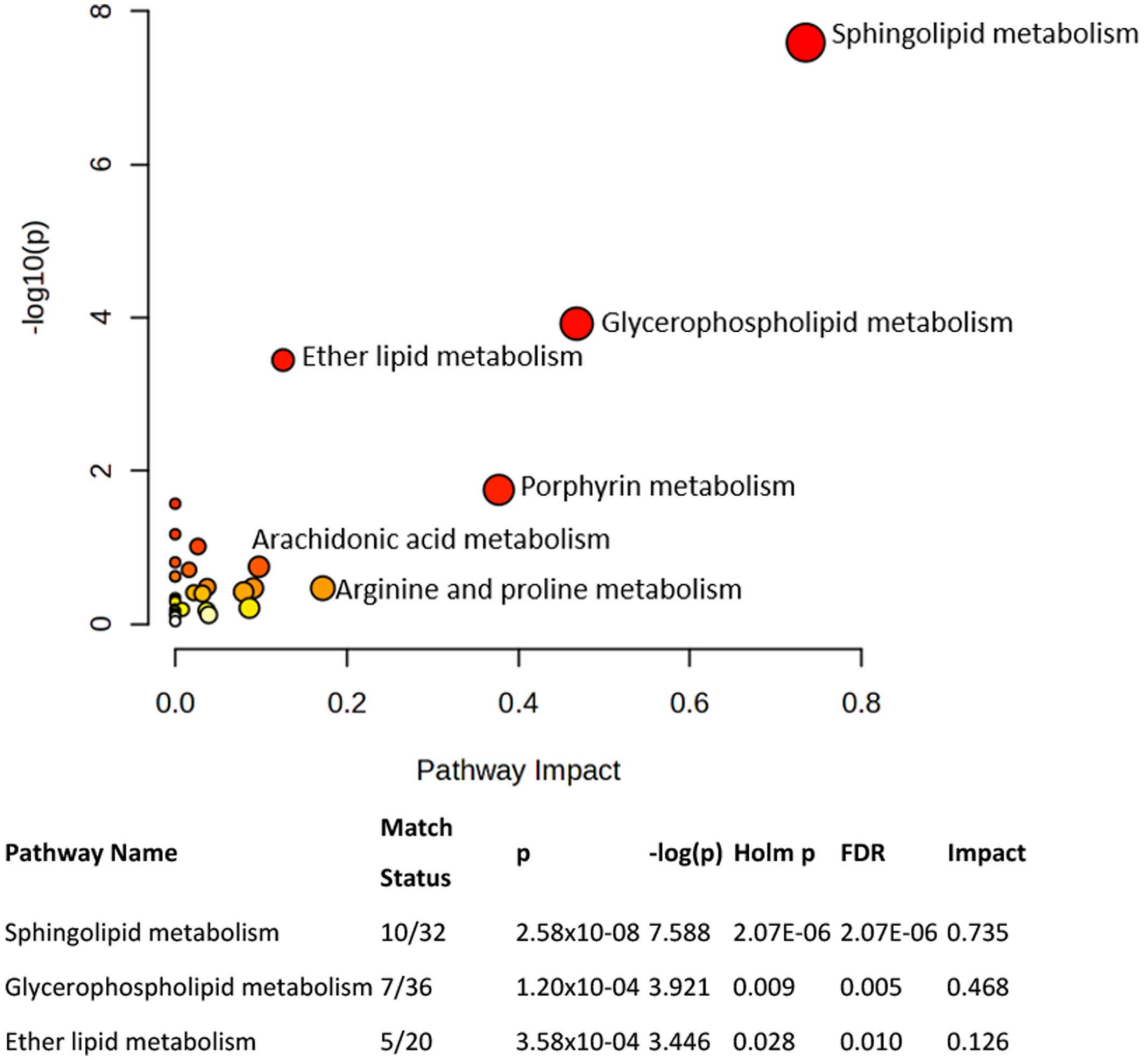
Pathway enrichment analysis associated with vitamin D status in the metabolomics sub-study. The bubble plot summarizes pathway enrichment results based on annotated endogenous metabolites in the metabolomics sub-study, comparing vitamin D–deficient versus non-deficient participants. The *y*-axis shows −log10(*p* value) for pathway enrichment, and the *x*-axis indicates pathway impact derived from topology analysis. Bubble size reflects the number of matched metabolites within each pathway, and color intensity indicates statistical significance. The most enriched lipid-related pathways were sphingolipid metabolism (10/32 matched metabolites; Holm-adjusted *p* = 2.07 × 10^−6^; FDR = 2.07 × 10^−6^; impact = 0.735), glycerophospholipid metabolism (7/36; Holm-adjusted *p* = 0.009; FDR = 0.005; impact = 0.468), and ether lipid metabolism (5/20; Holm-adjusted *p* = 0.028; FDR = 0.010; impact = 0.126).

Pathway enrichment analysis based on 565 annotated endogenous metabolites identified significant differences in three lipid-related pathways (Figure 2). Sphingolipid metabolism showed the strongest enrichment, with 10 of 32 matched metabolites differing by vitamin D status (Holm-adjusted *p* = 2.07 × 10^−6^; FDR = 2.07 × 10^−6^) and the highest topological impact score (0.735). Glycerophospholipid metabolism was also enriched (7/36 matched metabolites; Holm-adjusted *p* = 0.009; FDR = 0.005; impact = 0.468). Ether lipid metabolism showed moderate enrichment (5/20 matched metabolites; *p* = 3.58 × 10^−4^; Holm-adjusted *p* = 0.028; FDR = 0.010) with a lower topological impact score (0.126).

### Metabolite panel performance for vitamin D status classification

**Figure 3:**
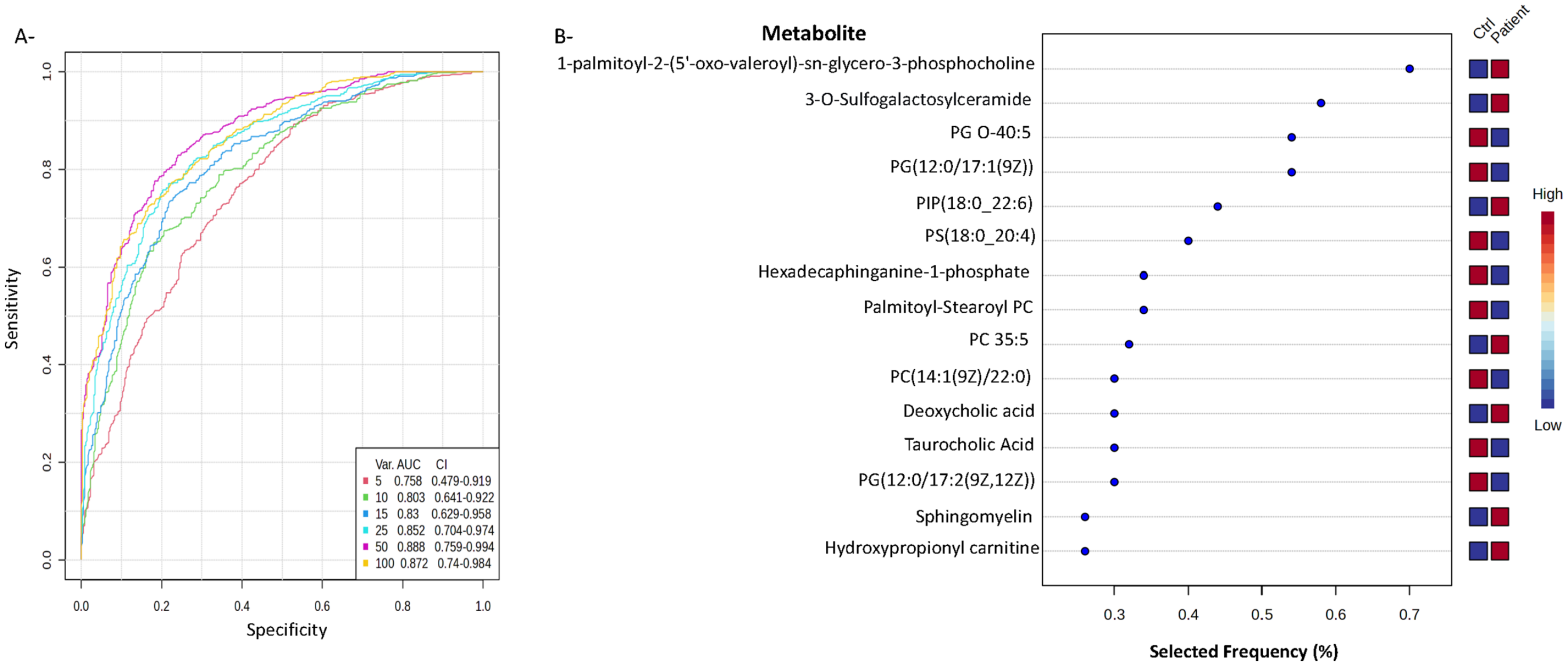
ROC analysis and feature selection frequency for classification of vitamin D status in the metabolomics sub-study (*n* = 50). **(A)** ROC curves evaluating classification of vitamin D status (deficient vs. non-deficient) in the metabolomics sub-study (*n* = 50). AUCs (95% CI) were 0.758 (0.479–0.919) for 5 features, 0.803 (0.641–0.922) for 10 features, 0.830 (0.629–0.958) for 15 features, 0.852 (0.704–0.974) for 25 features, 0.888 (0.759–0.994) for 50 features, and 0.872 (0.740–0.984) for 100 features, with the highest discrimination observed at 50 features. **(B)** Selection frequency plot for the top 15 discriminatory annotated metabolites/features; the *x*-axis indicates the proportion of model iterations in which each feature was selected.

ROC analysis showed improved discrimination of vitamin D status as the number of selected features increased, reaching optimal performance at 50 features (Figure 3A; AUC = 0.888). A model based on the top 15 features also demonstrated good discrimination between vitamin D–deficient and non-deficient participants, with 83% classification accuracy (Figure 3B).

## Discussion

Our findings support a strong association between vitamin D status and *H. pylori* infection in Lebanese adults. In both univariate comparisons and multivariable modeling, lower circulating 25(OH)D levels were consistently linked to infection status (Tables 1, 2). This observation is particularly relevant in Lebanon and the wider region, where hypovitaminosis D remains common despite abundant sunlight and is influenced by behavioral and environmental determinants that limit UVB exposure (4, 10–12). Notably, vitamin D deficiency is highly prevalent in Lebanon; in a large retrospective analysis of 19,452 adults, 31% were vitamin D deficient and 28% were insufficient (4), underscoring the clinical relevance of determinants and consequences of low serum 25(OH)D levels in this setting. Within this context, identifying correlates of vitamin D deficiency that intersect with infection risk has clear public health relevance.

Iron status and micronutrient depletion are often discussed in relation to *H. pylori*. In our cohort, mean serum iron level was lower among *H. pylori*–positive participants (Table 1), which is consistent with the pathophysiology of chronic *H. pylori* gastritis and reduced iron bioavailability (5). Systematic evidence also indicates that *H. pylori* infection is associated with depleted iron stores and that eradication with iron supplementation can improve hematologic indices in many settings (5). However, iron status (deficient vs. normal) was not independently associated with infection in our multivariable model (Table 2), suggesting that in this cohort the vitamin D–infection association is not simply a proxy for iron deficiency.

Sex differences were observed in multivariable analysis, with female participants showing lower odds of infection (Table 2), consistent with meta-analytic evidence of modest male predominance in adult *H. pylori* prevalence (13, 14). Importantly, for a vitamin D–focused interpretation, sex differences in serum 25(OH)D levels are highly context-dependent and reflect supplement use, sun-exposure behaviors, and cultural practices (11, 12, 15). Thus, sex may operate both as a demographic correlate of infection and as a determinant of vitamin D status, reinforcing its role as a key covariate in observational models examining 25(OH)D levels and infectious outcomes (11–15).

Season of sampling was included in both the univariate and multivariable analyses (Tables 1, 2, respectively), and was not significantly associated with *H. pylori* infection status in either analysis, which is consistent with the established understanding that *H. pylori* acquisition does not follow a seasonal pattern.

Vitamin D status was the most robust factor associated with *H. pylori* infection in our multivariable analysis: compared with vitamin D deficiency, both insufficiency and normal vitamin D status were associated with substantially lower odds of infection (Table 2). This aligns with the broader literature reporting inverse associations between 25(OH)D levels and *H. pylori* prevalence and/or eradication failure (16–19). Mechanistically, vitamin D signaling may influence mucosal host defenses through vitamin D receptor (VDR)-mediated innate immune pathways and antimicrobial effector responses (including cathelicidin-related mechanisms), which have been proposed to contribute to susceptibility and eradication outcomes in *H. pylori* infection (18, 19). Although our cross-sectional design precludes causal inference, the strength and consistency of the association observed in this cohort support the clinical value of assessing and correcting vitamin D deficiency as part of comprehensive care. However, vitamin D replacement should not be viewed as a substitute for standard antibiotic-based *H. pylori* eradication therapy, as recommended in the Maastricht VI/Florence Consensus Report (20) and the first regional Middle East consensus (21). The causal effects of vitamin D supplementation on infection outcomes remain to be prospectively confirmed (18, 19).

Beyond direct antimicrobial effects, vitamin D also contributes to immune regulation and intestinal homeostasis through activation of the vitamin D receptor (VDR) (22). VDR signaling supports epithelial barrier integrity, antimicrobial peptide expression, modulation of gut microbiota composition, and suppression of pro-inflammatory pathways (22–24). These mechanisms are relevant because altered VDR signaling and microbiome disruption have been linked to inflammatory, metabolic, and systemic disease risk (24, 25). Therefore, vitamin D deficiency may influence host inflammatory responses partly through effects on gut microbial balance and intestinal barrier function.

Lifestyle factors can be interwoven with both vitamin D status and infection risk. Physical activity intensity differed by infection status in Table 1, while physical activity (yes/no) showed only a borderline association in multivariable analysis (Table 2). This is compatible with evidence that physical activity alone does not reliably increase serum 25(OH)D levels once UVB exposure and related behaviors are accounted for, and that sun exposure patterns, seasonality, and sun-protective behaviors can dominate vitamin D variability in cross-sectional data (10, 26). In Middle Eastern populations, limited skin exposure and indoor lifestyle contribute to persistent hypovitaminosis D despite abundant sunshine, and clothing practices can markedly influence 25(OH)D levels (11, 12). Together, these considerations highlight why vitamin D deficiency may cluster with broader lifestyle/environmental factors that also shape infection risk, and why careful adjustment and longitudinal designs are needed to clarify the direction of causality. Bivariate and multivariable analyses may yield different results because they address different statistical questions. In our study, marital status was significantly associated with *H. pylori* infection status in the bivariate analysis (Table 1), but this association was attenuated and no longer significant after adjustment in the multivariable model (Table 2), suggesting confounding by other covariates. The same principle applies to sex: although sex distribution did not differ significantly in the unadjusted comparison (Table 1), female sex was associated with lower odds of infection after multivariable adjustment (Table 2). In contrast, coffee consumption was not significantly different in the unadjusted comparison, but was associated with higher odds of infection in the adjusted model.

For marital status, bivariate analyses in both the full cohort and the metabolomics sub-study showed a similar pattern, with lower *H. pylori* prevalence among unmarried participants and higher prevalence among married participants. However, after multivariable adjustment, neither married nor divorced status remained independently associated with *H. pylori* infection. This suggests that the unadjusted association may be partly explained by confounding or by household-related exposure patterns. Intrafamilial *H. pylori* transmission, including transmission between partners, is well documented (27); however, because the present study did not enroll couples or assess the infection status of household contacts, this potential transmission pathway could not be evaluated and is acknowledged as a limitation.

Our untargeted metabolomics approach revealed that vitamin D deficiency is associated with differential abundance of metabolites statistically enriched in lipid-related pathways, including sphingolipid, glycerophospholipid, and ether lipid metabolism. Because the vitamin D metabolomics analysis was not adjusted for *H. pylori* infection status, these metabolic signatures should be interpreted as exploratory vitamin D-associated findings, potentially confounded by infection status. These findings are biologically plausible: vitamin D signaling has been shown to modulate sphingolipid homeostasis and ceramide-related profiles, and disruption of this regulatory axis under vitamin D deficiency could alter sphingolipid-mediated immune and inflammatory signaling (6, 28).

Consistent with this, previous metabolomics studies indicated that *H. pylori* eradication is associated with changes in lipid metabolites, including sphingolipids and glycerophospholipids (29). Additionally, alterations in phosphatidylcholine species have been reported in several gastric diseases (30). Taken together, these findings suggest that vitamin D deficiency may be associated with altered lipid metabolic pathways that affect mucosal immunity and infection-related phenotypes; however, these observations warrant replication in larger studies with adjustment for infection status.

The pathway enrichment analysis (Figure 2) also identified alterations in porphyrin, arachidonic acid, and arginine/proline metabolism. Although these pathways were not among the most strongly enriched findings, they may still reflect broader biological differences associated with vitamin D status. Porphyrin metabolism is linked to heme biosynthesis and iron utilization (31), which may be relevant given the reduced serum iron level observed in our cohort. Arachidonic acid metabolism is central to the production of prostaglandins and leukotrienes that modulate inflammatory and immune responses in the gastric mucosa (32). Arginine and proline metabolism may also be relevant in this context because arginine contributes to nitric oxide synthesis and antimicrobial host defense, whereas proline metabolism is involved in collagen turnover, mucosal defense (33), and gastric mucosal regeneration (34). Alterations in this pathway may therefore indicate broader differences in host defense and mucosal repair associated with vitamin D status in the setting of *H. pylori* infection.

Oxylipins are bioactive lipid mediators derived from the enzymatic or non-enzymatic oxidation of omega-3 and omega-6 polyunsaturated fatty acids (35, 36). They regulate vascular tone, cell signaling, inflammation, and inflammation resolution, making them relevant to inflammatory and metabolic diseases (35, 36). In this context, vitamin D-related pathways have been associated with linoleic acid-derived oxylipins and isoprostanes (37), while *H. pylori* infection has been linked to alterations in omega-3 and omega-6 fatty acid metabolism (38), which provides substrates for oxylipin biosynthesis. Thus, oxylipin-related pathways may represent a plausible mechanistic link between vitamin D status, *H. pylori* infection, and host inflammatory-metabolic responses. However, because oxylipins were not directly quantified in the present study, this interpretation remains exploratory and should be evaluated in future targeted lipidomics studies.

Vitamin D-mediated antimicrobial defense may also involve cathelicidin pathways. Vitamin D promotes cathelicidin expression (39), while LL-37/hCAP18 has been implicated in gastric epithelial defense against *H. pylori* (40). More recent experimental work has also explored LL-37-based antimicrobial strategies against *H. pylori* (41). Therefore, vitamin D deficiency may theoretically impair cathelicidin-mediated mucosal protection, although LL-37/hCAP18 was not measured in this study.

This study has several limitations. The cross-sectional, retrospective design limits causal inference and is susceptible to residual confounding. The study was conducted at a single center, and 25(OH)D levels were measured at a single time point; however, the season of sampling was included as a covariate in the multivariable model.

Because the metabolomics analysis was not adjusted or stratified according to *H. pylori* infection status, the findings should be interpreted as exploratory associations with vitamin D status within a mixed *H. pylori* infection subset. This analysis does not allow distinction between metabolic differences independently related to vitamin D status and those potentially influenced by *H. pylori* infection. A robust stratified metabolomics analysis would require additional samples and repetition of the mass spectrometry experiment with adequate subgroup representation.

Targeted validation of key candidate metabolites in a larger cohort would be an important next step. In addition, LL-37/human cationic antimicrobial peptide 18 (hCAP18) levels were not measured; future studies should assess this pathway to clarify whether vitamin D deficiency impairs cathelicidin-mediated mucosal defense against *H. pylori* and related gastric inflammatory or carcinogenic mechanisms (40, 41), while also considering broader cancer-related molecular and biomarker pathways (42).

Despite these limitations, the work integrates cross-sectional epidemiologic association testing in the parent cohort (*n* = 502) with metabolomics-based pathway exploration in a subset (n = 50) and provides a vitamin D-centered rationale for prospective studies in Lebanon and similar settings.

## Conclusion

Vitamin D deficiency was strongly associated with *H. pylori* infection in this Lebanese cohort. In an exploratory metabolomics sub-study, vitamin D deficiency was associated with differences in lipid-related pathways, including sphingolipid, glycerophospholipid, and ether lipid metabolism. Larger prospective and mechanistic studies are needed to determine whether improving vitamin D status influences *H. pylori* susceptibility or eradication outcomes, to clarify whether the observed lipid signatures are independent of infection status, and to better define how lifestyle and environmental factors contribute to *H. pylori* infection risk.

## Data Availability

The raw data supporting the conclusions of this article will be made available by the authors, without undue reservation.
