# Supplementary material for: Vitamin D status and Helicobacter pylori infection: clinical associations and lipid pathway differences in an exploratory metabolomics sub-study
Source: Front Nutr. 2026 Jun 1;13:1812874. doi: 10.3389/fnut.2026.1812874 (PMC13265471; doi:10.3389/fnut.2026.1812874)
Supplement: Supplementary file 1 [file Supplementary_file_1.docx]

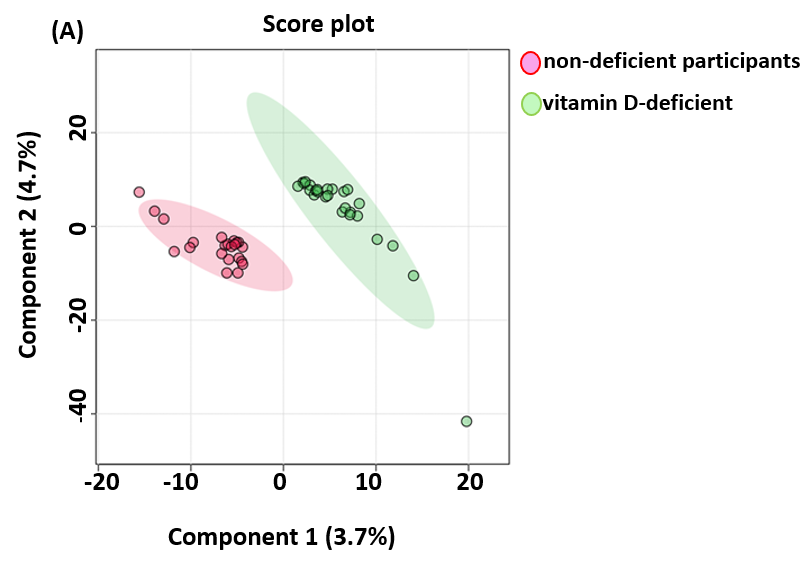

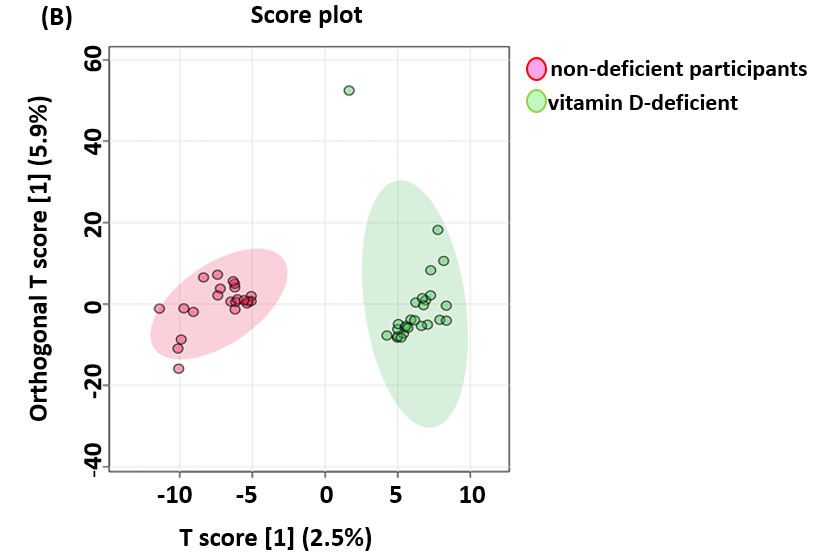


**Supplementary Figure 1.** Supervised multivariate discriminant analysis according to vitamin D status.

(A) PLS-DA score plot and (B) OPLS-DA score plot comparing vitamin D-deficient and non-deficient participants in the metabolomics sub-study.

**
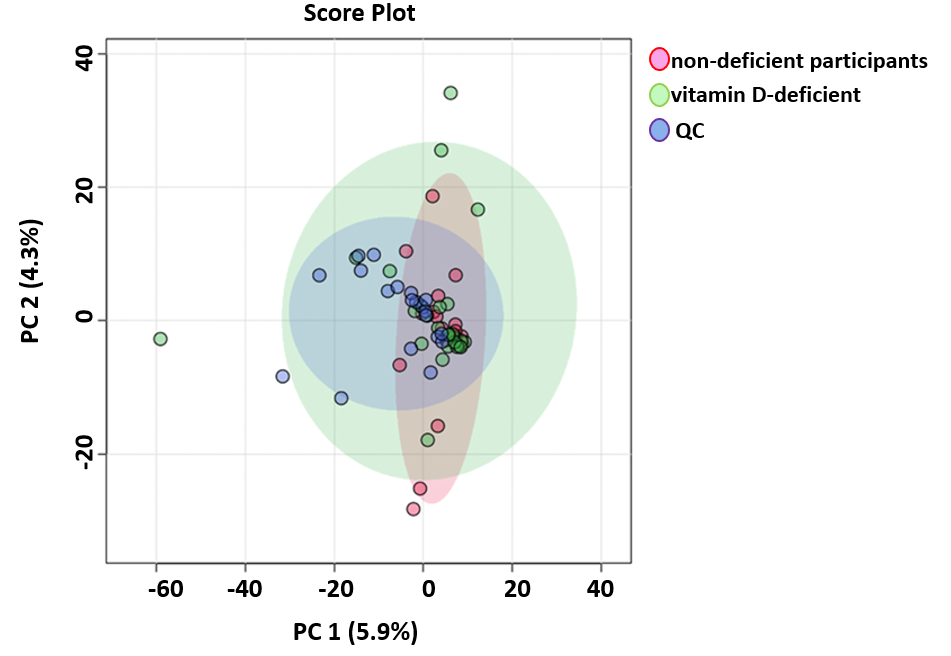
**

**Supplementary Figure 2. Principal component analysis according to vitamin D status and quality-control samples.**
PCA plot showing vitamin D-deficient samples, non-deficient samples, and quality-control samples. Quality-control samples clustered tightly in the central region, supporting analytical reproducibility. PC1 and PC2 explained 5.9% and 4.3% of the total variance, respectively. Ctrl, normal vitamin D levels; QC, quality control.

**Supplementary Table 1**: Baseline characteristics of participants included in the untargeted metabolomics sub-study.

|  | **Subset Characteristics (n=50)** |
| --- | --- |
| **Age (years)** | 35.44±14.68 |
| **Gender n (%)** |  |
| Males | 16 (32%) |
| Females | 34 (68%) |
| **BMI Categories n (%)** |  |
| Normal | 23 (46%) |
| Overweight and Obese | 27 (54%) |
| **Marital Status n (%)** |  |
| Not Married | 23 (46%) |
| Married | 24 (48%) |
| Divorced | 3 (6%) |
| **Cigarette Consumption n (%)** |  |
| Non-Smoker | 41 (82%) |
| 1- 10/ day | 3 (6%) |
| 11-20/ day | 6 (12%) |
| More than 20/ day | 0 (0%) |
| **Sleeping Hours n (%)** |  |
| Less than 7 hours | 20 (40%) |
| 7 hours or more | 30 (60%) |
| **Intensity of Physical Activity n (%)** |  |
| None | 27 (54%) |
| Light | 12 (24%) |
| Moderate | 6 (12%) |
| Vigorous | 5 (10%) |
| **Coffee Consumption n (%)** |  |
| No | 17 (34%) |
| Yes | 33 (66%) |
| Iron Level (µg/dL) | 79.30±38.05 |
| Serum 25(OH)D (ng/mL) | 20.65±11.55 |
